# Supplementary figures and images for: Ten-Second Cold Water Stress Test Differentiates Parkinson’s Disease from Multiple System Atrophy: A Cross-Sectional Pilot Study
Source: Biomedicines. 2025 Jun 28;13(7):1585. doi: 10.3390/biomedicines13071585 (PMC12292825; doi:10.3390/biomedicines13071585)

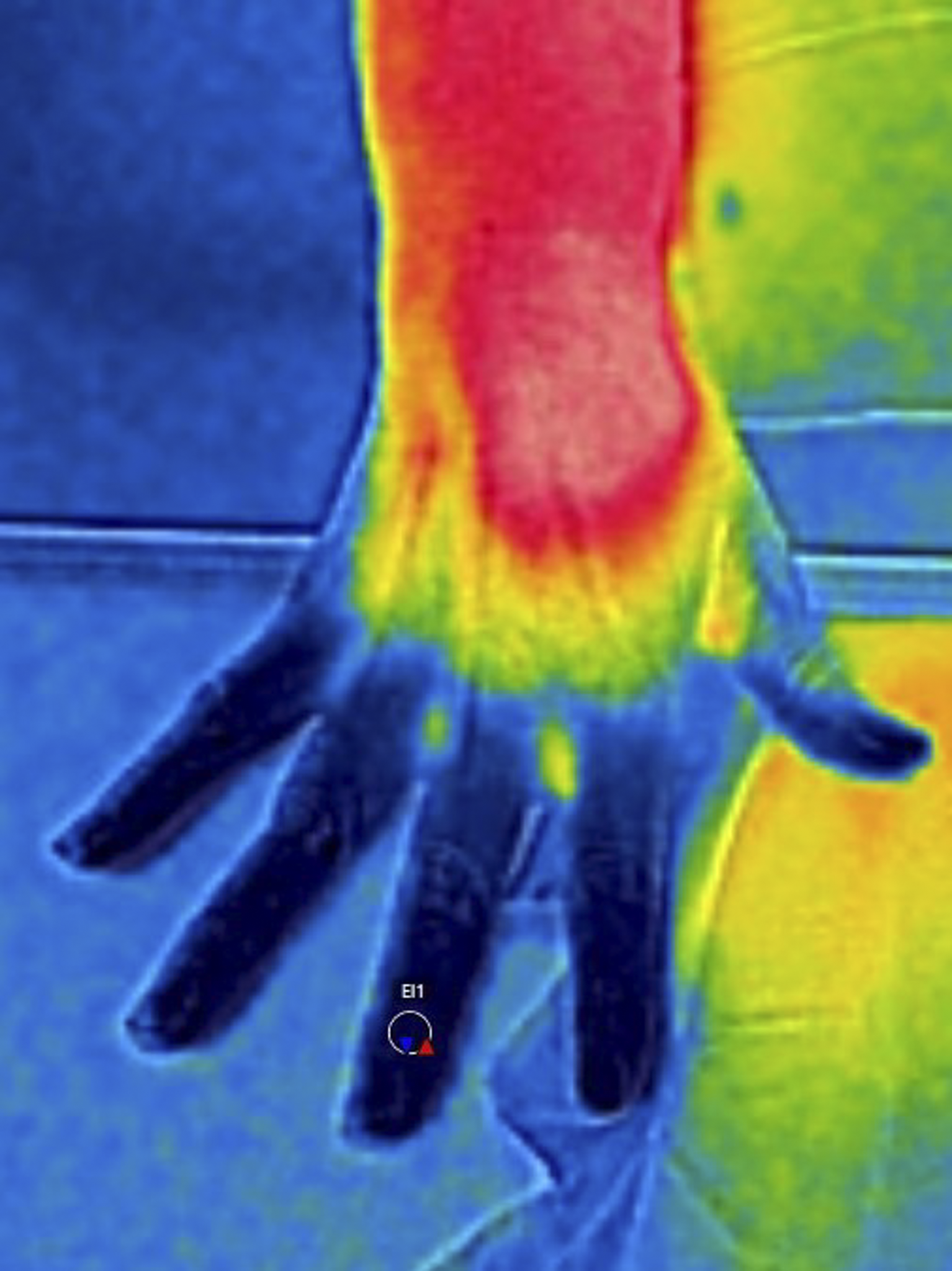

Supplement: Supplementary file 1 [file biomedicines-13-01585-s001.zip › supple figure 1.tif]

Supplemental Figure 2A

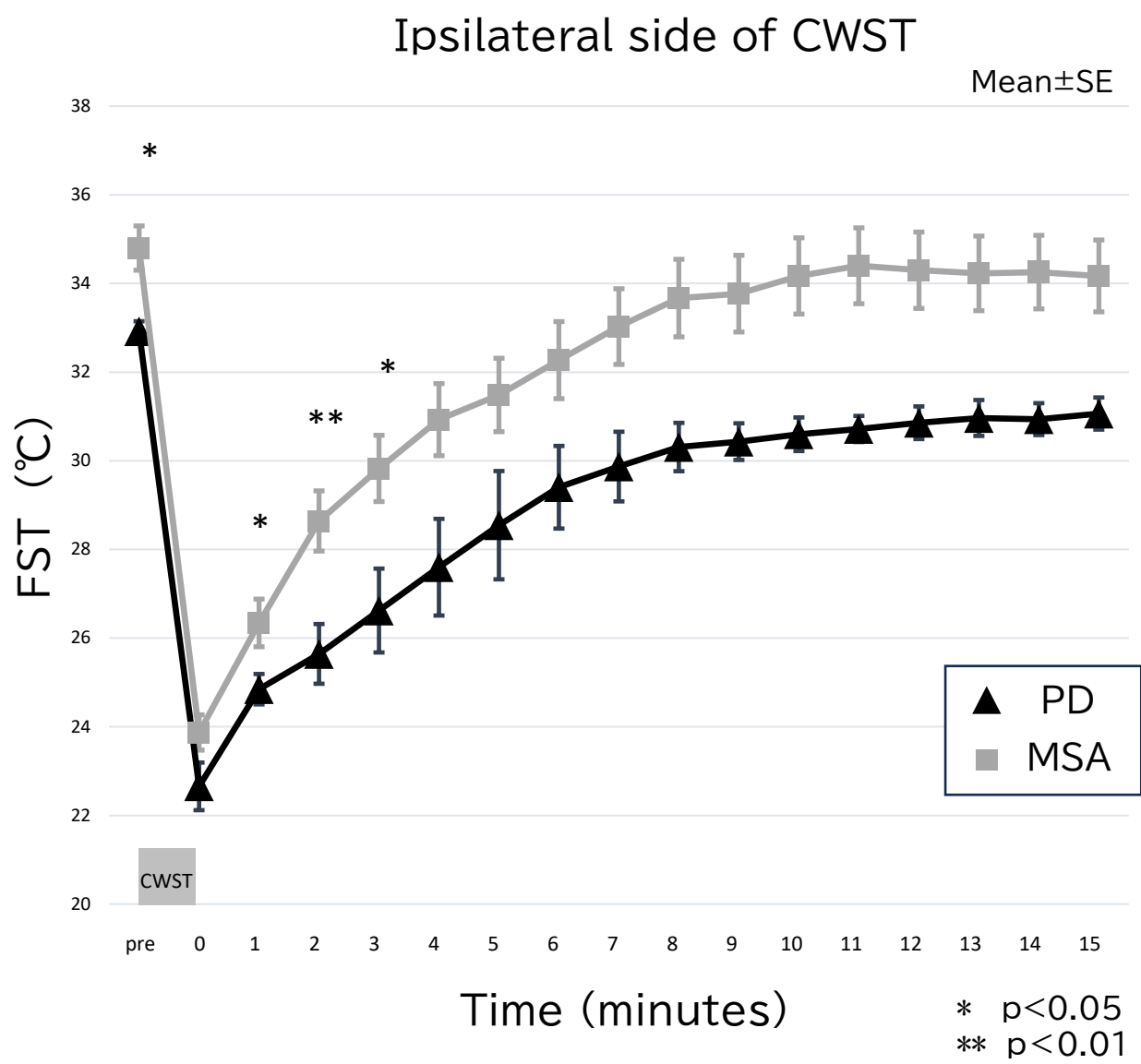

Supplemental Figure 2B

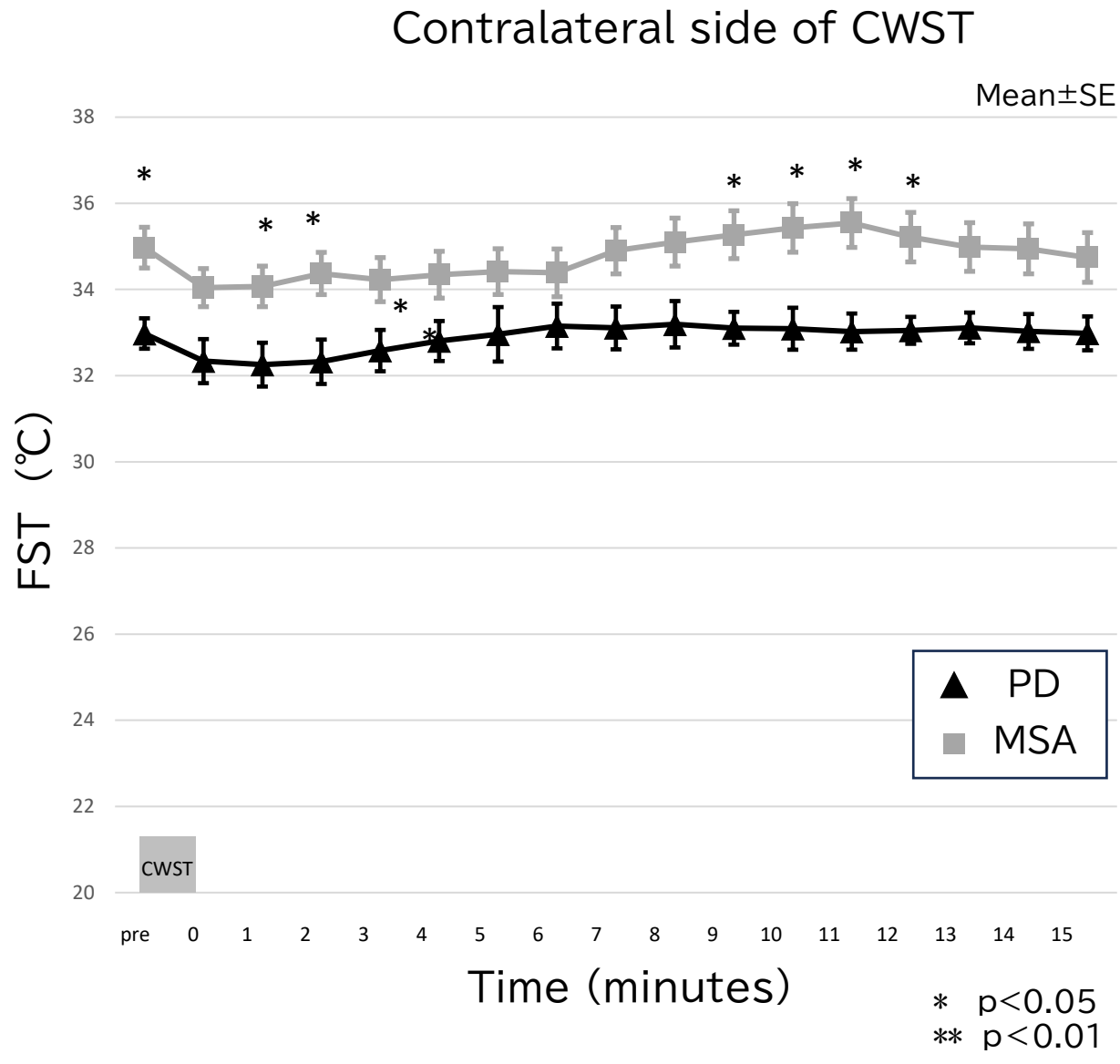

Supplement: Supplementary file 1 [file biomedicines-13-01585-s001.zip › Supple_figure_2.pdf]
